# The Role of Regulatory Myeloid Cell Therapy in Renal Allograft Rejection

**DOI:** 10.3389/fimmu.2021.625998

**Published:** 2021-02-24

**Authors:** Jingming Zhuang, Jiangang Hou

**Affiliations:** Department of Urology, Huashan Hospital, Fudan University, Shanghai, China

**Keywords:** kidney transplant, regulatory myeloid cell, allograft rejection, immunosuppression, prevention, end-stage renal disease

## Abstract

Kidney transplantation is a primary therapy for end-stage renal disease (ESRD) all the time. But it does not mean that we have fully unraveling the mystery of kidney transplantation and confer every patient favorable prognosis. Immune rejection has always been a stumbling block when we try to increase the success rate of kidney transplantation and improve long-term outcomes. Even if the immune rejection is effectively controlled in acute phase, there is a high possibility that the immune response mediated by chronically activated antibodies will trigger chronic rejection and ultimately lead to graft failure. At present, immunosuppressive agent prepared chemically is mainly used to prevent acute or chronic rejection, but it failed to increase the long-term survival rate of allografts or reduce the incidence of chronic rejection after acute rejection, and is accompanied by many adverse reactions. Therefore, many studies have begun to use immune cells to regulate the immune response in order to control allograft rejection. This article will focus on the latest study and prospects of more popular regulatory myeloid cells in the direction of renal transplantation immunotherapy and introduce their respective progress from experimental research to clinical research.

## Introduction

In 1954, Dr. Merril and Murray of Harvard University completed the first successful kidney transplant between a pair of twins in order to avoid allograft immunity. The patient did not take any immunosuppressive drugs, and the transplanted kidney achieved long-term survival (1). But this is a rare condition after all, and there are certain genetic differences between most donors and recipients, mainly referring to human leukocyte antigen (HLA), which greatly ascends the possibility of allograft rejection (2). Based on the onset time, the rejections that occur after kidney transplantation can be classified into hyperacute rejection reaction, accelerated rejection reaction, acute rejection reaction, and chronic rejection reaction. The most important ones are acute rejection and chronic active antibody-mediated rejection (AMR). We are going to discuss the prevention and treatment of them (3). Thanks to preoperative histocompatibility tests, more reasonable and standardized surgical procedures, improved tissue typing, and especially the advent of securer and more efficient immunosuppressive drugs, the incidence of rejection (mostly acute rejection) during the first year has gradually decreased over the years (4). Clinical kidney transplant has become the gold standard for ESRD, which improves patients' quality of life within a certain period compared with continuous dialysis (3, 5). At present, the main clinical method of chemical immunosuppressive treatment routinely after kidney transplantation is calcineurin inhibitor (CNI), and other drugs including mycophenolic acid and corticosteroids are usually used in combination (6, 7). Immunosuppressive agents seem to have reached their limits, but many problems remain, such as leading to excessive suppression of immune system and increasing the rate of infectious diseases and the incidence of tumors (8). It can also cause side effects without an immunological relationship, including nephrotoxicity, making recipients susceptible to cardiovascular dysfunction, metabolic diseases, and complications of other organs (9). Unfortunately, due to the lack of immunological or antigen specificity, CNI based immunosuppressive can hinder alloresponses, but does not ultimately prevent late dysfunction or loss. And in fact, CNI treatment even causes inhibition of regulatory T-cell development, which leaves a major challenge (10). Therefore, although these drugs reduce the rate of acute rejection, they fail to significantly improve the long-term survival of transplanted kidney (11).

Given the concerns over chemical immunosuppressive agents and the disappointing long-term results of kidney transplants, it is clearly necessary to revisit our choice of immunomodulation methods. Although immune rejection is mainly mediated by effector T cells or antibodies secreted by B cells, some other types of white blood cells can promote a tolerant immune response and extend survival of the graft (12). In kidney transplant immunity, mechanisms of tolerance are crucial because whether an allograft is acceptable to the recipient depends largely on the balance between effector cells with alloantigen reactivity and regulatory immune cells (13). Regulatory immune cells are one such group of leukocyte populations that have the potential to specifically prevent acute rejection or graft-vs.-host disease (GVHD). They gradually acquire regulatory functions during development, and the immunosuppressive property is acquired in the local microenvironment of allografts or in lymphatic tissue drained by the graft. And this immunosuppressive property will cause the surrounding environment to change toward the long-term survival of the graft, thereby preventing the graft from being rejected or GVHD. Therefore, regulatory immune cells play a critical role in influencing long-term outcomes after kidney transplantation (14).

## Regulatory Myeloid Cells

**Figure 1:**
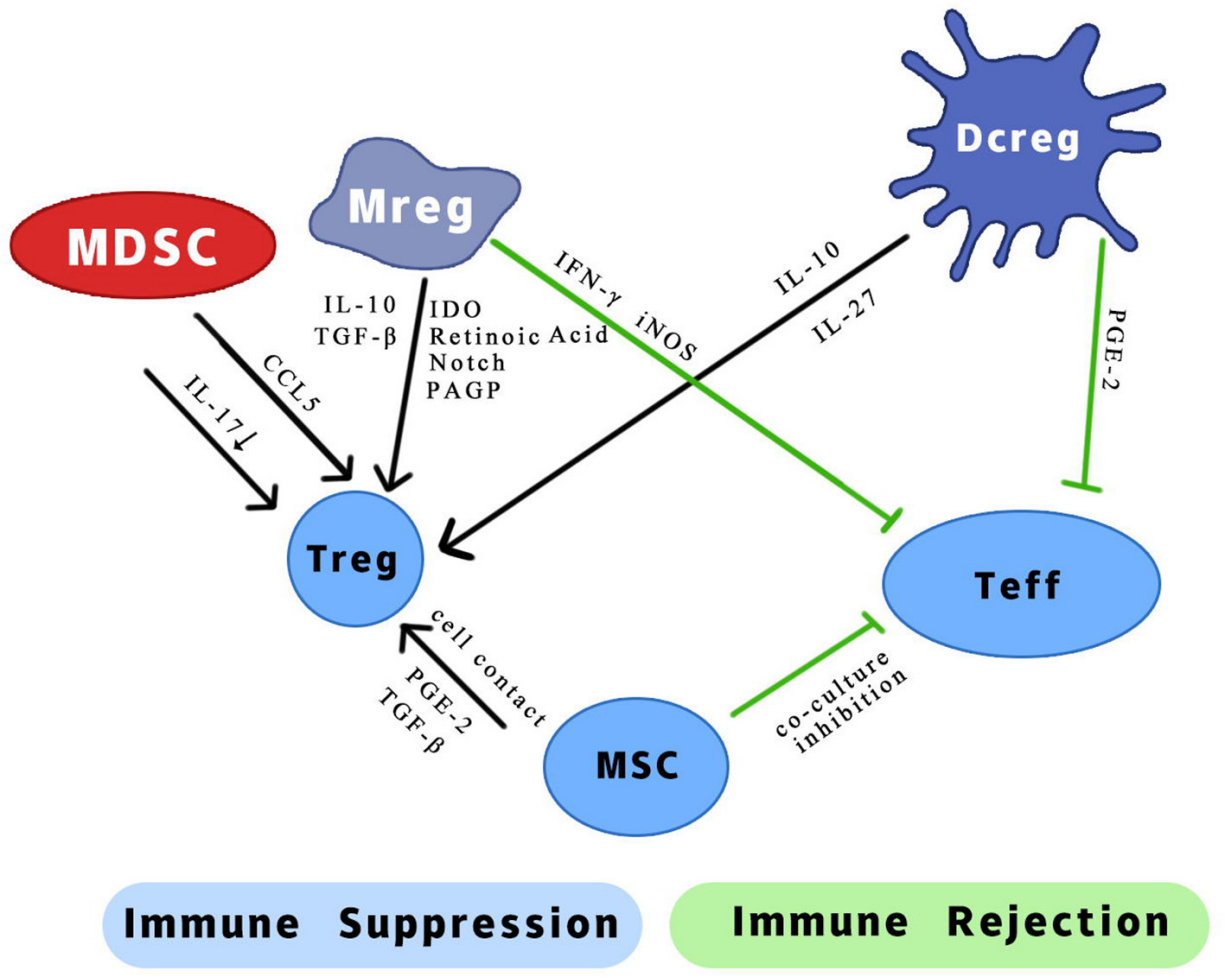
Possible mechanism of different effects of regulatory myeloid cells on two types of T cells. IL, interleukin; CCL5, chemotactic C-C motif 5; IFN-γ, interferon-gamma; iNOS, inducible nitric oxide synthase; TGF-β, transforming growth factor-β; IDO, indoleamine 2,3-dioxygenase; PAGP, progestagen-associated endometrial protein; PGE-2, prostaglandin E2.

Currently, researchers working on organ transplantation are hoping to minimize the dependence of recipients on immunosuppressive drugs and increase donor-specific immune tolerance using immunomodulatory therapy. Many animal experiments and observations have shown that regulatory immune cells can promote transplant tolerance and reduce the infection rate after kidney transplantation (15). There are also considerable experiments confirming that they could prolong the survival of kidney allografts in non-human primate (NHP) models (16, 17). Most of these experiments focus on the role of regulatory T cells (Tregs). From basic experiments to clinical related research, Tregs have excellent immune regulation effect after transplantation of various organs, and they occupied an important position in transplantation immunity (13, 18, 19). There was some small-scale clinical trials which purify Tregs from patients' own blood and perform polyclonal expansion. Experimental results show that the project is safe and well-tolerated. The infused Tregs have a certain degree of durability and stability, proven to reduce the inflammation of the transplanted kidney through biopsy (20). Another group of cells with regulatory functions, regulatory myeloid cells, has excellent immunosuppressive effects in many transplant responses, in addition to being more diverse and complex. Some of these effects received widespread attention because they may be related to Tregs (Figure 1). The regulatory myeloid cells discussed in the previous articles generally include three types, which are regulatory macrophages, regulatory dendritic cells, and myeloid-derived suppressor cells (21). Due to the homology and excellent anti-rejection prospects, here we will broadly include bone marrow mesenchymal stromal cells. We will focus on the progress of kidney transplantation immunity of several regulatory myeloid cells, and their characteristics are shown (Table 1).

**Table 1:** Feature of regulatory myeloid cell (22–25).

**Regulatory myeloid cell**	**Source**	**Growth factor**	**Immunophenotypic markers**
Regulatory macrophages	Rodent BM cells or human PBMC	M-CSF, IFN-γ	CD14^low^ CD16^−/*low*^ CD80^low^ CD83^−/*low*^ CD86^+/hi^ HLA-DR^+/hi^
Regulatory dendritic cells	Rodent BM cells or human PBMC	GM-CSF,IL-4 Tolerogenic Factor(s):IL-10, TGF-β, VitD3, Dexamethasone, rapamycin	MHC II ^low^ CD40^low^ CD80^low^ CD86^low^ PDL1^hi^ TGF-β^hi^ IL-10^hi^
Myeloid-derived suppressor cells	Rodent BM cells or human PBMC	Activation:IL-1β, IL-6, IL-13, PGE-2, TNF-α, VEGF Expansion: G-CSF, GM-CSF	Three subpopulations (22)
Mesenchymal stromal cells	Extraction from BM	^−^	CD117^−^ CD31^−^ CD44^+^ CD29^+^ Sca-1^+^

*BM, bone marrow; PBMC, peripheral blood mononuclear cell; M-CSF, macrophage colony stimulating factor; IFN-γ, interferon-γ; GM-CSF, granulocyte-macrophage colony stimulating factor; TGF-β, transforming growth factor-β; PDL1, programmed cell death ligand-1; PGE-2, prostaglandin E2; TNF-α, tumor necrosis factor-α; G-CSF, granulocyte colony stimulating factor*.

## Regulatory Macrophages

Macrophages are derived from monocytes and they are an important part of innate immunity. When the body is injured, monocytes in the blood vessels are transported to the inflamed tissue and become macrophages. The accumulation of macrophages in organs has been considered a feature of allograft rejection for many years (26). Depending on the microenvironment, immunogenic monocytes will infiltrate into the allograft early after transplantation, and be renamed macrophages, which will respond to the transplanted organ and trigger organ rejection (27). The classically activated macrophages' (M1) inflammatory response is characterized by high levels of pro-inflammatory cytokines in the surrounding environment and the promotion of the Th1 response. However, in addition to the role of M1 in promoting immune responses, alternatively activated macrophages (M2) are believed to be associated with the alleviation of tissue inflammation. The latest research found that when macrophage colony-stimulating factor (M-CSF) and interferon-γ (IFN-γ) act on monocytes, they will transform into a new type of inhibitory cells called regulatory macrophages (Mregs). Mregs are another uniquely characterized group of cells that can produce interleukin 10 (IL-10) but do not generate arginase-1, which can reduce the proinflammatory immune response (28). Recent studies have shown that Mregs promote successful long-term transplantation and may be necessary for inducing transplant tolerance (29). Hutchinson et al. administered Mregs to two living donor kidney transplant recipients. After transplantation, two patients were treated with the smallest dose of tacrolimus monotherapy (aiming for trough serum levels between 4 and 8 ng/ml) in 24 weeks and maintained favorable transplant function later. No adverse effects were observed in 2 patients, with 3 years follow-up (30). It is inferred that this phenomenon may be caused by Mregs inhibiting the proliferation of effector T cells by producing IFN-γ. During cell experiments, it was found that mouse Mregs can produce induced nitric oxide synthase (iNOS) to inhibit the activity of T cells *in vitro* and can kill and engulf co-cultured T cells subsequently (31). The latest research also shows that Mregs can convert allogeneic CD4 + T cells into regulatory T cells, thereby non-specifically inhibiting bystander T cells and the maturation of dendritic cells. Preoperative administration of donor-sourced Mreg to kidney transplant recipients can cause a sharp increase in circulating Treg, promoting the acceptance of allogeneic transplantation by rapidly inducing Tregs (32). In addition to conducting further clinical and *in vitro* experiments, some researchers have proposed the concept of macrophage isolation and characterization according to the tissue environment, and the function of macrophages according to different circumstances (33). Du et al. conducted *in vitro* and *in vivo* experiments and found that insulin-like growth factor 2 (IGF-2) can induce anti-inflammatory phenotype of mature macrophages by changing their mitochondrial metabolism, and transfer them to mice can alleviate their disease (34). Maybe using IGF-2 or other inducing factors can also actively induce macrophages to produce specific cells with Mreg function based on their phenotypes for application in immune regulation after kidney transplantation.

## Regulatory Dendritic Cells

Dendritic cells (DCs) are another type of cells that are essential to elicit a specific T cell response to alloantigen, but they can also enhance a tolerance response in some cases (35). DCs are functionally classified as myeloid DCs (mDCs) and plasmacytoid DCs (pDCs). Since most of them belong to mDCs, they are generally classified as myeloid immune cells. DCs belong to important antigen presenting cells (APCs), which play an essential role in the activation and adjustment of non-specific and specific immunity and induction of immune tolerance to maintain homeostasis in steady state under inflammatory conditions. Although the main function of DCs is to trigger the immune system, it seems that both mDCs and pDCs can enhance the tolerance to the same antigen (36). The two-way role of DCs in coordinating this delicate balance has attracted widespread attention. Increasing evidence indicates that the coordinate ability of DCs is dependent upon their immature state and can be regulated by many factors such as immunosuppressive molecules, genetic mechanism, certain pathogenic stimulation, signals from immune cells or apoptotic cells, and tissue or tumor microenvironment (37). Regulatory DCs (DCregs) retain the ability to present antigens to specific T cells. However, at the same time, they descend the expression of costimulatory molecules (CD80, CD86, and CD40) and pro-inflammatory cytokines (IL-12), and ascend the expression of inhibitory molecules (PDL1, CD95L, and IDO) and anti-inflammatory cytokines (TGF-β and IL-10), and is resistant to signals that induce maturation (38). The mechanisms of DCregs promoting immune tolerance include inducing T cell non-responsiveness, generating Tregs, inhibiting T cell response and inducing T cell apoptosis (39). The result published by Marin E et al. shows that DCregs regulate immune responses through lactate synthesis, inhibition of T cell proliferation and expansion of Tregs by secreting factors. The high level of lactate shifts T cell response to tolerance, delaying graft-vs.-host disease (40). At the University of Nantes, as part of the ONE Study, autologous DCreg infusion 1 day before transplant, is under examination in renal transplantation with SOC triple immunosuppressive (azathroprine, steroid, tacrolimus) (41). Therefore, researchers have begun to consider how to efficiently prepare DCregs to facilitate future large-scale use. Cai et al. produced enough high-quality functional DCregs from induced pluripotent stem cells (iPSCs) derived from murine, and named them iPS-DCregs. Even under strong stimuli, this kind of DCreg can still be in a “stable immature stage,” and can induce permanent acceptance of mouse heart allografts (42). Some researchers have successfully used mouse DCregs to perform allogeneic transplantation of mouse skin and significantly prolonged graft survival (43). Pang et al. went a step further, purifying exosomes from DCregs and injecting them into allogeneic kidney transplantation model mice. After observation, it was determined that the exosomal group significantly improved survival, reduced the levels of (CD4+) T cell and cytokines related to rejection (IFN-γ, IL-2, and IL-17), and promoted the percentage of (Foxp3+) (CD4+)T cells in mice allograft, which indicates that exosomes of DCregs are involved in the induction of immune tolerance (44). Recently, Thomson et al. have produced Good Manufacturing Practice (GMP) grade DCregs for human organ transplantation. They briefly reviewed their experience in modulating immunotherapy in organ transplantation and the generation and characterization of human monocyte-derived DCregs. And they proposed a phase I/II safety study in which the effect of donor-derived DCregs combined with traditional immunosuppressive agent treatment on subclinical and clinical allograft rejection and adverse reactions will be observed in detail (45).

## Myeloid-Derived Suppressor Cells

Myeloid derived suppressor cells (MDSCs) are a heterogeneous population of bone marrow-derived myeloid progenitors that suppress immune responses in different kinds of inflammatory environments, such as organ transplantation, malignant tumors, infectious diseases, and autoimmune diseases (46). They are functionally defined as a class because of phenotypically expression with characteristics related to precursors of hematopoietic cells that can stably differentiate into mature macrophages, DCs and granulocytes at all stages (47). The pivotal position of MDSCs was initially established in the field of tumor immunity (48). Since then, many researchers have begun to explore the role of MDSCs in the host's immune response during organ transplantation. Dugast et al. first reported the important role of MDSCs in inducing tolerance in rat kidney transplant models. By increasing the number of MDSCs, the proliferation of allogenic T cells can be inhibited *in vivo*, and MDSCs may participate in the phase of NO-dependent tolerance maintenance (49). After this groundbreaking study determined the importance of MDSCs in transplant immunity, research on the therapeutic potential and immunoregulatory effects of MDSCs has been extensively conducted. Currently, MDSC are divided in three subpopulations: polymorphonuclear (PMN), monocytic (M) and early-stage (e) MDSC, and each one has different immunosuppressive mechanism (22). Dilek et al. focused on the connection between MDSCs and Tregs in transplant immunity and discovered the importance of a chemotactic C-C motif 5 (CCL5) between the two. The results show that the gradient of CCL5 around the graft contributes to MDSC's enhancement of tolerance in kidney allograft recipients. This gradient controls the recruitment of Tregs to the graft, which may help maintain tolerance (50). Studies performed by Luan et al. revealed blood derived M-MDSCs were able to expand Treg *in vitro* and correlated with increased Treg numbers *in vivo* (51). There are also many studies focusing on the effect of using MDSCs to extend the survival time of transplants after allogeneic transplantation of organs such as heart and skin in mice (52–55). These studies have discovered pathways and mechanisms such as carbon monoxide that may be related to the immune suppression of MDSCs through animal experiments, but more teams have begun to turn more to clinical trials. Meng et al. compared the allogeneic function, severity of tissue damage, and long-term survival of patients with high and low MDSCs *in vivo* after kidney transplantation. Compared with the low-dose MDSCs group, the allograft function of the high-dose MDSCs group was significantly enhanced. In addition, his team also found that MDSCs isolated from transplant recipients can up-regulate Tregs and inhibit IL-17 production *in vitro* (56). Hock et al. found that the number of MDSCs in patients after kidney transplantation increased rapidly and reached a peak after the start of immunosuppression, but then fluctuated due to unknown reasons. Therefore, the purpose of establishing follow-up research is to explore the factors that regulate MDSC mobilization (57). Recent studies have shown that in patients with intestinal transplantation, MDSCs increase *in vivo* and restrain the response of effector T cells to the intestinal epithelium of the graft (58). This shows that due to the close relationship with T cells, MDSCs may have the prospect of immunosuppression for various organ transplants throughout the body. But some pessimistic points emerged, Lee et al. showed that adoptive MDSCs transfer after transplantation improves graft survival in mice, however their late depletion did not damage graft survival (59). Moreover, Utrero-Rico et al. found that higher maintained level of M-MDSC after transplantation in kidney recipients is associated with higher risk of cancer (60). These findings demonstrate together that MDSC could be beneficial early after transplantation to prolong graft survival, but, if maintained, it may promote tumoral development for patients due to excessive immunosuppression.

## Mesenchymal Stromal Cells

Bone marrow-derived mesenchymal stromal cells (MSCs) are also a type of cells that have regulatory functions in transplantation immunity. Although MSCs are found in many tissues, most are derived from bone marrow, as we also include them in regulatory myeloid cells. MSCs are a kind of cells with multi-directional differentiation potential. They can induce differentiation into a variety of tissue cells under specific *in vivo* and *in vitro e*nvironments (61). MSCs could also migrate to sites of inflammation and to transplanted organs. So, many researchers have put it into testing for autoimmune diseases and post-transplant immunomodulatory therapies (62, 63). In experiments aimed at post-transplant immunity, MSCs have shown unique, effective, and undisputed immune-modulating properties. MSCs can act on a wide range of immune cells such as T cells, B cells, macrophages, natural killer cells, dendritic cells, and granulocytes (64). The much profound impact of MSC on adaptive immune response may be closely related to its innate source, so myeloid cells may be the earliest affected cells. MSCs interact with innate and acquired immune cells and immunomodulate them to make them develop more stable (and occasionally more intense) (65, 66). They impede the differentiation of dendritic cells and inhibit their maturation, thereby downregulating the ability of antigen presenting cells *in vivo* and *in vitro* (67–69). MSCs reduce the cytotoxicity of T lymphocytes that respond to foreign antigens and prevent effector T cells from replicating at the G0/G1 phase, thereby reducing their ability to produce IFN-γ and IL-2 (70). And they also promoted T cells to differentiate into Tregs and induce graft tolerance (71, 72). In addition, MSCs alter the proliferation of natural killer cells and γδT cells (73), reduce cytotoxicity and IFN-γ production, and inhibit B cell activation and antibody secretion (74).

Although there are many studies in various fields, the exact mechanism of action of MSCs has not been obtained. However, since MSCs can be easily isolated from different tissues including bone marrow, umbilical cord or connective tissue, and based on the current advanced technology, MSCs can be expanded *in vitro* to achieve an applicable cell dose in a relatively short time for clinic (75). Many studies on the use of MSCs cell therapy to modulate immunity after kidney transplantation have been performed worldwide. The goal of these studies is usually to induce inhibition through various forms of treatment with MSCs, so that patients can maintain the effect of immune tolerance based on minimal doses of immunosuppressive drug therapy to get the long-time survival of graft (76).

## Conclusion

From the above analysis, it can be found that the immune system has a natural balance. This balance consists of the coordination of pro-inflammatory and regulatory cells, which involves a large series of regulatory factors. This is a very complicated and delicate process. As an important part of the regulatory cell side and possessing capability of promoting Treg proliferation, regulatory myeloid cells are expected to enhance success of early immunosuppressive drug withdrawal and become one of the anti-rejection options for patients after renal transplantation soon. An interesting matter to remark would be the need of prospective studies regarding the regulation of myeloid regulatory cells on the long term and which is the effect of immunosuppressive regiments on these cells, as well as their relationship with clinical outcomes, in order to develop strategies to promote tolerance. Most pilot studies were highly variable in design, did not incorporate a parallel trial arm of patients. This requires us all to further carry out more clinical-based experiments based on researches that has been reproduced in order to prove the advantages of its efficacy and higher safety and operability. In addition, whether it can be made into the very promising and available cell-based medicinal products (CBMP) also requires a unified standard. The existing data is still very ambiguous on this point.

## Author Contributions

JZ collected data, performed the analysis, and prepared the manuscript. JH conceived the article contents and endorsed the final draft submitted. Both authors contributed to the article and approved the submitted version.

## Conflict of Interest

The authors declare that the research was conducted in the absence of any commercial or financial relationships that could be construed as a potential conflict of interest. The reviewer RR declared shared affiliation, with no collaboration, with the authors to the handling editor at the time of review.
